# Mortality among Methadone Maintenance Clients in China: A Six-Year Cohort Study

**DOI:** 10.1371/journal.pone.0082476

**Published:** 2013-12-12

**Authors:** Xiaobin Cao, Zunyou Wu, Li Li, Lin Pang, Keming Rou, Changhe Wang, Wei Luo, Wenyuan Yin, Jianhua Li, Jennifer M. McGoogan, for the National Methadone Maintenance Treatment Program Working Group

**Affiliations:** 1 National Center for AIDS/STD Control and Prevention, Chinese Center for Disease Control and Prevention, Beijing, China; 2 Semel Institute Center for Community Health, University of California Los Angeles, Los Angeles, California, United States of America; 3 Yunnan Institute of Drug Abuse, Kunming, Yunnan, China; Johns Hopkins School of Public Health, United States of America

## Abstract

**Objective:**

To assess the overall mortality of methadone maintenance treatment (MMT) clients in China and its associated factors.

**Methods:**

A total of 1,511 MMT clients, all of whom enrolled in China's first eight MMT clinics between March and December 2004, were included in this cohort study and followed for approximately six years, until June 2010. Mortality and its predictors were examined using Cox proportional hazards regression models.

**Results:**

A total of 154 deaths were observed within 5,391 person-years (PY) of follow-up for an all-cause mortality rate of 28.6 per 1,000 PY. The leading causes of death were drug overdose (33.8%), HIV/AIDS-unrelated disease (21.4%), and HIV/AIDS (16.9%). The all-cause mortality rate of clients engaged in MMT for one year or less was roughly three times that of clients who stayed in MMT for four years or more (14.0 vs. 4.6, p<0.0001), HIV-positive subjects was nearly four times mortality rate than that of HIV-negative individuals (28.1 vs.6.8, p<0.0001). ART-naive HIV-positive subjects had approximately two times higher mortality rate than those receiving ART (31.2 vs. 17.3, <0.0001). After adjusting for confounding variables, we found that being male (HR = 1.63, CI: 1.03–2.57, p = 0.0355) and being HIV-positive (HR = 5.16, CI: 3.70–7.10, p<0.0001) were both associated with higher risk of death whereas increased durations of methadone treatment were associated with a lower risk of death (HR = 0.26, CI: 0.18–0.38, p<0.0001 for two to three years, HR = 0.08, CI: 0.05–0.14, p<0.0001 for four or more years).

**Conclusion:**

Overall mortality was high among MMT clients in China. Specific interventions aimed at decreasing mortality among MMT clients are needed. Our study supports the need for keeping client at MMT longer and for expanding ART coverage and suggests the potential benefits of integrated MMT and ART services for drug users in China.

## Introduction

Drug users (DUs) are well-known to have a much higher risk of death than non-DUs [1]–[2], with most likely causes of death being overdose, suicide, and HIV/AIDS-related disease [3]–[5]. As China is now thought to have the largest DU population in the world, some 2.2 million [6], its drug use epidemic has become a major public health concern. Furthermore, the high-risk behavior of injecting DUs was the initiator and the major driver of the early HIV epidemic in China. By the end of 2011, approximately 780,000 people were estimated to be living with HIV/AIDS nationwide, with 28.4% of infections attributed to injecting drug use and HIV prevalence among DUs as high as 50% in some regions [7].

In an attempt to combat the growing drug abuse and HIV/AIDS epidemics, China's National Methadone Maintenance Treatment (MMT) Program was piloted in 2004, scaled up starting in 2006, and as of April 2012, encompassed 747 clinics nationwide, covering more than 350,000 DUs, cumulatively. Several studies have since documented the positive impacts of this program among DUs on illicit drug use, HIV incidence, and quality of life, as well as other factors [8]–[12]. Furthermore, evidence from MMT programs in other countries document lower mortality rates among MMT clients as compared to DUs not in treatment [13]–[16]. Among DUs in MMT, mortality rates in other settings range from 2.4 per 1,000 PY to 14.9 per 1,000 PY [17]–[18], and factors found to be associated with improved survival include high methadone dose, long duration of MMT retention, and antiretroviral therapy (ART) for those who were HIV-positive [19]–[20]. However, mortality among DUs in China's MMT program has thus far not been assessed.

Therefore, the purpose of this study was to examine the overall mortality of MMT clients enrolled in the first eight MMT clinics in China, and examine factors associated with improved survival in this setting. We sought to test the hypothesis that while mortality is high among clients in China's MMT program, increased length of methadone treatment is associated with improved survival.

## Methods

### Study design

**Figure 1:**
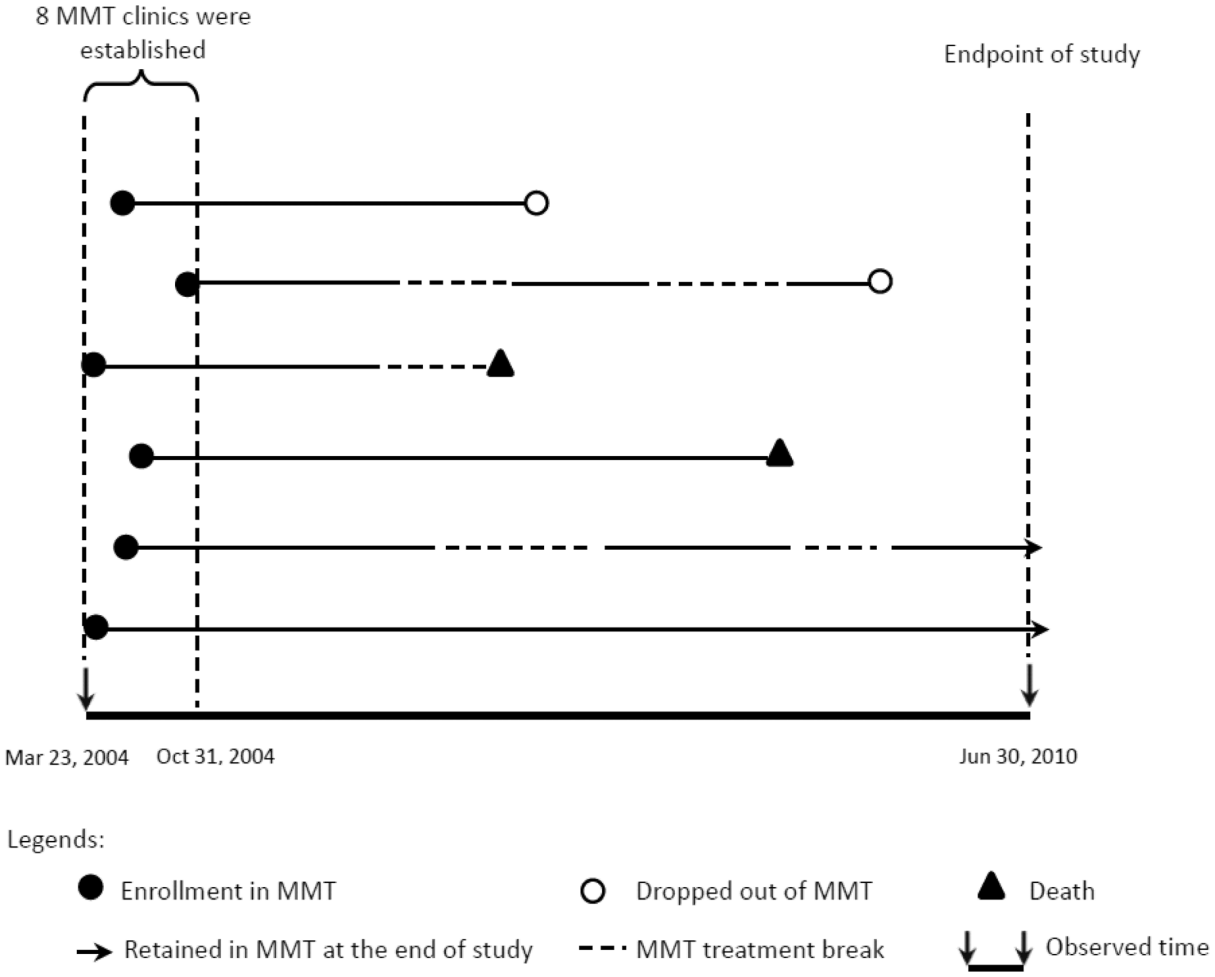
The overall picture of clients' treatment dynamics. The study participants were enrolled between March 23, 2004 and October 31, 2004, and followed until June 30, 2010. Treatment break means clients missed more than 30 consecutive days during the follow-up period. The first line (solid line) means a client was treated continuously before he/she dropped out of MMT. The second line means a clients dropped out of MMT for a period of time (broken line), reenrolled in MMT (solid line), and then dropped out of MMT again. Even if a client dropped out of MMT, he/she was followed so he/her (live or dead) was assessed during the follow-up period.

The China national MMT program is actually a prospective, observational open cohort of treatment of opioid users. This study is a secondary analysis of existing government MMT data. A subset of fixed cohort MMT clients enrolled in the first 8 clinics between 23 March 2004 and 31 December 2004, were selected and followed up to 30 June 2010. The mortality of participants in the first eight MMT clinics in China (two in Sichuan, one in Yunnan, two in Guizhou, one in Guangxi, and two in Zhejiang) was assessed from 23 March 2004 to 30 June 2010, and verified retrospectively in 2010. The dynamic nature of the cohort is presented as in **Figure 1**. The cohort was followed in a unique environment with a centralized and nationwide data system - China's MMT Information Management System. This facilitated accurate ascertainment of date and cause of death for all subjects.

### Study subjects

**Figure 2:**
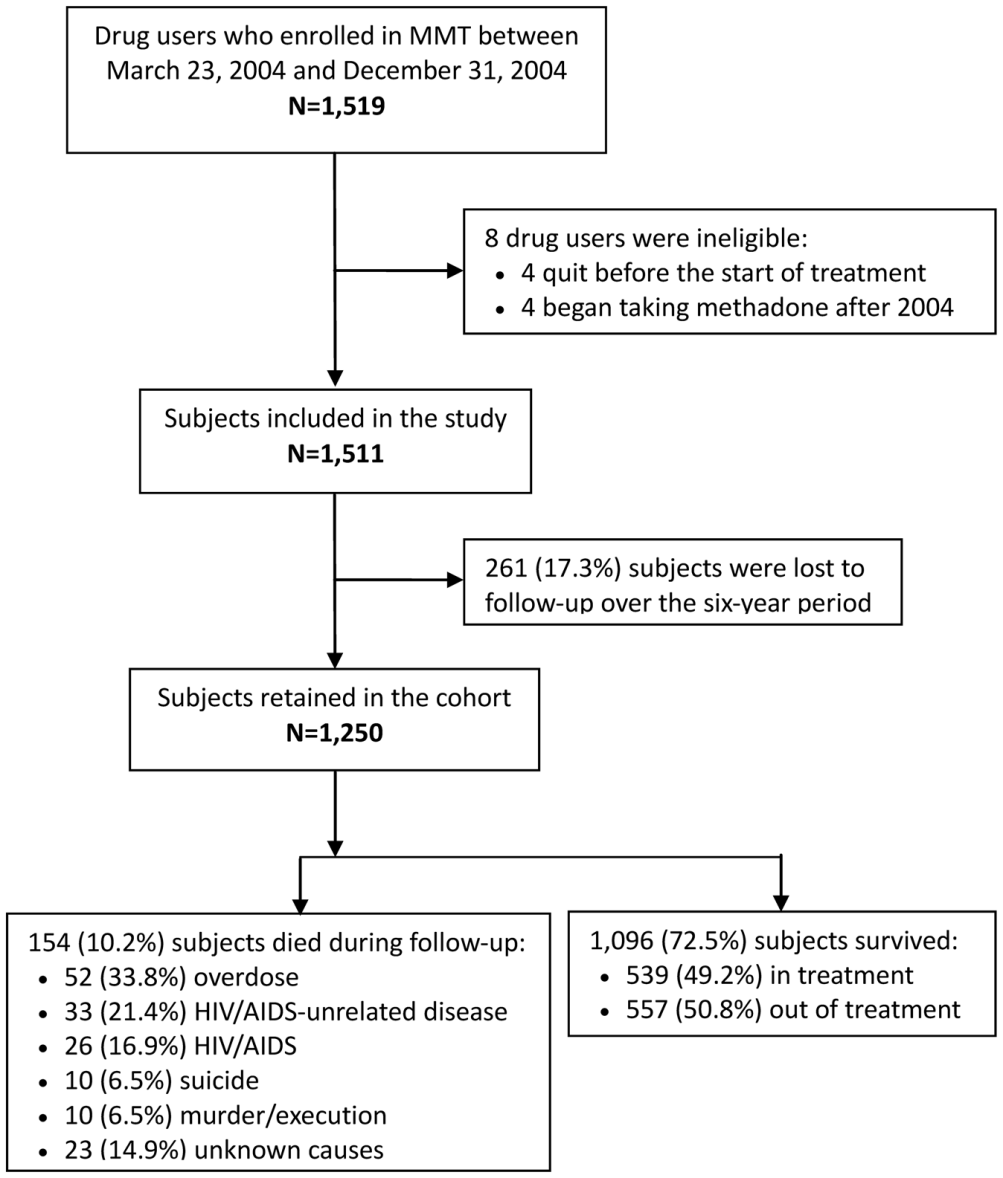
Flow diagram depicting the development of the study cohort as well as causes of death for deceased subjects and in- or out-of-treatment status for surviving subjects.

When the national MMT program began in China, all participants were required to meet the following MMT program enrollment eligibility criteria: 1) have failed several times at attempts to discontinue heroin use, 2) have spent at least two terms in a compulsory detoxification center, 3) be 20 years old or older, 4) be registered as a local resident in the area in which the clinic is located, and 5) be of full civil capacity. HIV-positive DUs were only required to meet the latter two criteria [8]–[9]. The study subjects for this study were clients of MMT clinics who were enrolled in China's national MMT program between 23 March 2004 and 31 December 2004. A total of 1,511 MMT clients were included in our study cohort. A flow diagram depicting the development of our cohort is shown in **Figure 2**.

### Data collection

China's MMT Information Management System was launched simultaneously with the opening of the first eight MMT clinics in 2004. This system is used to record and retain MMT client data and has since been upgraded to a web-based system in 2008 [21]. Each patient is given a unique treatment number for tracking purposes in this system upon entry into the MMT program and each clinic uploads its daily services records to the system in real-time. Data collected include demographic information and drug use history on enrollment, daily methadone dose records, results of urine opiate tests and HIV and hepatitis C test results at entry, 6 months, 12 months, and then at 12-month intervals thereafter. In addition, if patients died or discontinued taking methadone for 30 consecutive days, special efforts were made to collect data on patients' death or drop-out reasons.

Socio-demographics and drug use history were collected in baseline and follow-up surveys, which were administered by trained interviewers in private rooms at MMT clinics within the first month of treatment and at the end of the study. Treatment experiences, including daily methadone dose, duration of methadone treatment, and urine tests positive for opiates during treatment, as well as dates and causes of death during the follow-up were extracted from MMT Information Management System. Due to the fact that methadone doses for each individual client tend to change over time, methadone exposure was expressed as the average methadone dose and calculated as the sum of doses taken divided by the cumulative number of days methadone was received. The proportion of urine tests positive for opiates during treatment was calculated as the number of positive urine opiate drug screen results divided by the total number of tests administered and expressed as a percentage. ART exposure was categorized using an intent-to-treat approach, whereby subjects were categorized as ART-treated if they had ever initiated ART, regardless of their level of adherence or duration of treatment.

Dates and causes of death were identified by MMT clinic or local centers for disease control and prevention (CDC) staff members by continuous on-site follow-up. In addition, dates and causes of death were ascertained from linkages with death records of MMT clinics and public security sectors as well as information provided by family members, which limited our lost to follow-up population and enabled subjects to be followed until their death or the end of study period even if they discontinued MMT. Causes of death were determined based on the International Statistical Classification of Diseases Codes Version 10 [22].

### Statistical analysis

Because it's a prospective cohort study followed by approximately six years, survival analysis methods were used. Univariate analysis was conducted before proceeding to more complicated models. First, we descriptively analyzed the demographic, drug use history, and MMT treat characteristics of our study population. Second, for all categorical variables, Kaplan-Meier curves were created and log-rank tests were used to look at whether or not to include predictors in the final model. For continuous variables, they have been categorized then Kaplan-Meier curves and log-rank tests been used to find potential candidates for the final model. Variables found to have a p-value of less than 0.25 in univariate analysis and groups of variables found to be proportional were included in the final multivariate model. Third, Cox proportional hazards regression models were built to investigate predictors of mortality. We treated death at the end of the six-year period as the dependent variables and others as predictor variables. Dummy variables were created for categorical variables selected in the model. Hazard Ratios (HRs) and their 95% confidence intervals (CIs) were reported, all p-values were two-tailed and a p-value of less than 0.05 was considered to indicate statistical significance. All statistical analyses were performed using SAS software (version 9.2, SAS Institute, USA).

### Ethics Statement

This study was a secondary data analysis using existing Chinese government MMT program data. All MMT clients signed an informed consent upon enrollment to participate in MMT program saying that their data, after removing their personal identifications, could be used in epidemiological studies such as this. Therefore, no additional study-specific consent for this current study was sought. This study protocol was reviewed and approved by the Institutional Review Board of the National Center for AIDS/STD Control and Prevention, China CDC.

## Results

### Study population

A total of 1,511 subjects were enrolled in the study (Figure 2). Over the six-year follow-up period, a total of 154 subjects (10.2%) died during the six-year follow-up period. In our study population, 77.1% (1,165) were male, 77.8% (1,176) were unemployed, 60.3% (912) were aged 30 years old above, and 31.9% (482) reported having close family relationships upon enrollment. At enrollment, 67.2% (1,015) were injecting drug users, and 30.6% (337) did not have any contact with other drug users, whereas 19.5% (295) had at least 30 contacts with drug users in the past 30 days. Average age of onset of drug use was 23.8 years (SD = 5.7), average length of drug use was 8.6 years (SD = 3.3), average travel distance between the clinic and home was 5.5 kilometers (SD = 8.4), average daily dosage was 48.6 mg (SD = 32.4). As the requirement of China's MMT protocol, clients were required to do the urine test monthly when they stayed in the MMT program. In this study, the median of urine tests positive for opiates during treatment was 15.2% (1.8%, 40.0%).

### All-cause mortality and gender-specific standardization mortality rate

As summarized in Table 1, among the study cohort of 1,511 individuals, 10.2% (154) died during the study period. Male clients represented 85.7% (132) of these deaths. When causes of death were examined, 33.8% (52) were deemed due to overdose, 21.4% (33) due to non-HIV/AIDS related diseases, 16.9% (26) due to HIV/AIDS, 6.5% (10) due to suicide, and 6.5% (10) due to murder or execution. We further found that male patients were more likely to die than female patients (male *vs.* female: 24.5 *vs.* 13.0 per 1,000 PY) and that male clients were more likely to die due to overdose (male *vs.* female: 8.7 *vs.* 3.0 per 1,000 PY), non-HIV/AIDS related diseases (male versus female: 5.2 *vs.* 3.0 per 1,000 PY), and HIV/AIDS (male *vs.* female: 4.5 *vs.* 1.2 per 1,000 PY).

**Table 1:** All-cause mortality and gender-specific standardization mortality rate for the study population during six-year follow-up.

Causes of Death	All Clients	Male Clients		Female Clients	
	Deaths N (%)	Deaths N (%)	SMR per 1,000 PY	Deaths N (%)	SMR per 1,000 PY
Overdose	52 (33.8)	47 (35.6)	8.7	5 (22.7)	3.0
HIV/AIDS-unrelated disease	33 (21.4)	28 (21.2)	5.2	5 (22.7)	3.0
HIV/AIDS	26 (16.9)	24 (18.2)	4.5	2 (9.1)	1.2
Suicide	10 (6.5)	9 (6.8)	1.7	1 (4.5)	0.6
Murder/execution	10 (6.5)	9 (6.8)	1.7	1 (4.5)	0.6
Unknown	23 (14.9)	15 (6.8)	2.8	8 (36.4)	4.7
Overall	154 (100)	132 (85.7)	24.5	22 (14.3)	13.0

SMR: standardization mortality rate, PY: person-years.

### Mortality and its predictors

Table 2 presented the results of Log-rank tests of mortality rate by demographics, drug use history, and MMT treatment characteristics of clients on enrollment. The all-cause mortality rate of clients who remained in the MMT was almost the same as that of clients who dropped out MMT (10.6 vs. 10.0, p = 0.8333). The all-cause mortality rate for HIV-positive subjects was nearly four times that of HIV-negative individuals (28.1 vs.6.8, p<0.0001). Moreover, among HIV-positive subjects, those not receiving ART had approximately two times higher mortality than those receiving ART (31.2 vs. 17.3, p <0.0001).

**Table 2:** Comparisons of mortality rate by demographic and drug use history on enrollment and MMT treatment characteristics of clients.

Characteristics	Non-death	Death	Mortality rate	P-value
***Socio-demographics at time of MMT enrollment***				
Gender				
Male	1033	132	11.3	0.0043
Female	324	22	6.4	
Age (years)				
≤30	544	55	9.2	0.3168
>30	813	99	10.9	
Employment status				
Unemployed	1052	124	10.5	0.3462
Employed	305	30	9.0	
Living situation				
With family or friends	1062	117	9.9	0.1929
Alone	295	37	11.1	
Relationship to family				
Close	430	52	10.8	0.0959
Average	678	68	9.1	
Estranged	249	34	12.0	
HIV status				
Negative	1186	87	6.8	<0.0001
Positive	171	67	28.1	
Antiretroviral therapy (ART) **				
No	128	58	31.2	<0.0001
Yes	43	9	17.3	
Crime committed in past month				
No	110	26	19.2	0.0009
Yes	909	103	10.2	
Refused to answer/missing	338	25	6. 9	
				
***Drug use history at time of MMT enrollment***				
Length of drug use (years)			7.2	
≤6	362	28	10.1	0.0122
7 – 12	847	95	17.5	
≥13	146	31		
Drug use in past month (times per day)				11.1
1 – 2	209	26	9.2	0.1634
3 – 4	917	93	13.2	
>4	231	35		
Drug use by injection in past month				7.5
No	459	37	11.5	0.0032
Yes	898	117		
Needle sharing in past month			11.2	
No	688	87	9.1	0.6682
Yes	669	67		
Contacting current drug user in past month (total)				11.1
0	353	44	10.9	0.8824
1 – 30	539	66	10.2	
>30	265	30	18.0	
Refused to answer/missing	200	44		
***Post-enrollment MMT program participation***				
Average methadone dose (mg/day)*				
Low (≤30)	249	25	9.1	0.2035
Medium (31 – 60)	855	104	10.8	
High (>60)	253	25	9.0	
Remaining in MMT				
Yes	539	64	10.6	0.8333
No	818	90	10.0	
Distance from home to MMT clinic (km)				
<5	887	98	14.0	0.5192
≥5	624	56	9.5	
			4.6	
Relatives who were MMT clients				
No	1175	137	10.4	0.0489
Yes	199	17	7.9	
Urine tests positive for opiates during treatment (%)†				
≤10	708	84	10.6	0.0625
11 – 20	174	18	9.4	
≥21	475	52	9.9	
Overall	1357	154	10.2	

*Average methadone dose is calculated as the sum of all methadone doses divided by the number of days methadone was received.

**Only HIV-positive clients are included in the category “Antiretroviral therapy (ART).”

^†^Urine tests positive for opiates during treatment is calculated by the total number of routine urine drug tests that yielded positive results as a percentage of the total number of tests taken during the six years of follow-up.

**Figure 3:**
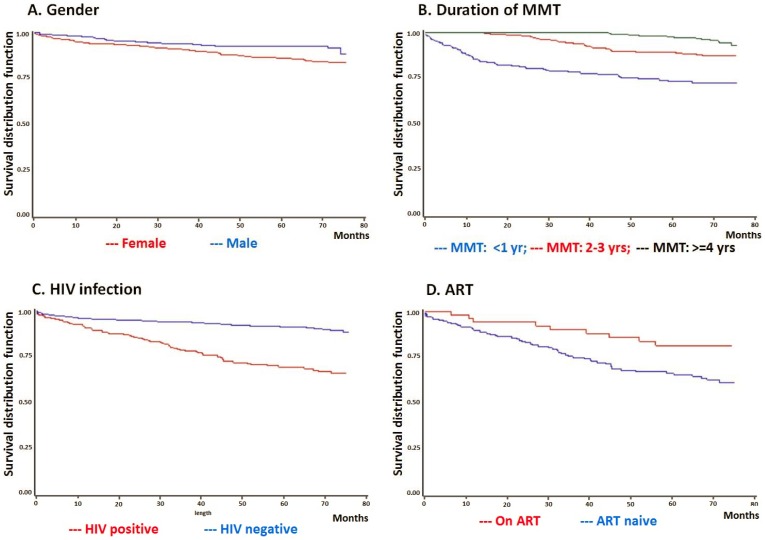
Kaplan-Meier curves by gender, length of MMT, HIV infection and ART.

Kaplan-Meier curves have been generated for all potential variables and selected curves are presented in Figure 3. According to results of Log-rank tests and the shapes of Kaplan-Meier curves, it was found that gender, living situation, injection in the past month, having relatives who were MMT patients, HIV status, ART and urine tests positive for opiates during treatment were included in the final Cox PH regression models. Meanwhile, length of treatment was included in the final Cox PH regression models. Table 3 summarized deaths, observed time, mortality rates and predictors of mortality over the six-year follow-up period. A total of 154 deaths were observed within 5,391.0 PY of follow-up for an all-cause mortality rate of 28.6 per 1,000 PY. The all-cause mortality rate of clients engaged in MMT for one year or less was roughly ten times that of clients who stayed in MMT for four years or more (81.1 per 1,000 PY vs. 8.2 per 1,000 PY, p<0.0001). The all-cause mortality rate for HIV-positive subjects was nearly four times that of HIV-negative individuals (71.4 per 1,000 PY vs. 19.5 per 1,000 PY, p<0.0001). Adjusting for confounding variables including living situation, injection in the past, having relatives who were MMT patients, we found that being male (HR = 1.63, CI: 1.03–2.57, p = 0.0355) and being HIV-positive (HR = 5.16, CI: 3.70–7.10, p<0.0001) were both associated with higher risk of death among our MMT client sample. By contrast, increased durations of methadone treatment were associated with a lower risk of death (HR = 0.26, CI: 0.18–0.38, p<0.0001 for two to three years, HR = 0.08, CI: 0.05–0.14, p<0.0001 for four or more years).

**Table 3:** Results from multivariate Cox PH models indicating factors associated with mortality over the six-year follow-up period.

Characteristics	Deaths, n	Observed Time in PY*	Mortality Rate per 1,000 PY§	HRs (95% CI)§	P-value&	Adjusted HRs (95% CI)§	P-value#
Gender							
Female	22	1304.0	16.9	1.00		1.00	
Male	132	4087.0	32.3	1.66 (1.04–2.64)	0.0346	1.63 (1.03–2.57)	0.0355
Living situation							
With family or friends	117	4345.3	26.9	1.00			
Alone	37	1045.7	35.4	1.42 (0.97–2.07)	0.0710		
HIV status							
Negative	87	4452.6	19.5	1.00		1.00	
Positive	67	938.5	71.4	5.39 (3.79–7.67)	<0.0001	5.16 (3.70–7.19)	<0.0001
Drug use by injection in past month							
No	37	1931.0	19.2	1.00			
Yes	117	3460.0	33.8	1.15 (0.78–1.69)	0.4763		
Average methadone dose (mg/day)†							
Low (≤30)	23	711.9	32.3	1.00		1.00	
Medium (31 – 60)	106	3456.4	30.7	0.94 (0.61–1.46)	0.7880	0.92 (0.59–1.43)	0.7067
High (>60)	25	1222.8	20.4	0.59 (0.33–1.04)	0.0669	0.64 (0.41–0.99)	0.0435
Length of treatment (years)#							
≤1	91	1122.4	81.1	1.00		1.00	
2 – 3	45	2071.1	21.7	0.27 (0.19–0.40)	<0.0001	0.26 (0.18–0.38)	<0.0001
≥4	18	2197.5	8.2	0.09 (0.05–0.15)	<0.0001	0.08 (0.05–0.14)	<0.0001
Relatives who were MMT clients							
No	137	4441.7	30.8	1.00			
Yes	17	949.3	17.9	1.14 (0.67–1.92)	0.6368		
Urine tests positive for opiates during treatment (%)‡							
≤10	84	2370.5	35.4	1.00		1.00	
11 – 20	18	875.1	20.6	0.60 (0.35–1.00)	0.049	0.59 (0.35–0.98)	0.0416
≥21	52	2145.4	24.2	0.44 (0.30–0.62)	<0.0001	0.43 (0.30–0.62)	<0.0001
Overall	154	5391.0		-		-	-

*Observed time in the study was calculated as the time from the start of methadone treatment to either death or study completion (30 June 2010), whichever occurred first.

§ PY: person-years, HR: hazard ratio, CI: 95% confidence interval.

† Average methadone dose is calculated as the sum of all methadone doses divided by the number of days methadone was received.

# Length of treatment is calculated as the total number of days that clients took methadone in the MMT clinics, than transfer to the number of years by dividing 365.

‡ Urine tests positive for opiates during treatment is calculated by the total number of routine urine drug tests that yielded positive results as a percentage of the total number of tests taken during the six years of follow-up.

# Adjusting for confounding variables including living situation, injection in the past month, having relatives who were MMT patients.

& Results of univariate Cox regression.

## Discussion

The purpose of this study was to test the hypothesis that mortality among China's MMT clients is high, but that longer MMT program retention is associated with improved survival. Therefore, we conducted a prospective cohort study among 1,511 DUs enrolled in China's national MMT program during its first nine months of operation at its original eight clinics, examining mortality and its associated factors over six years of follow-up.

We indeed found that the overall mortality rate of MMT clients in China was high, 28.6 per 1,000 PY, much greater than that found in Taiwan [17], Israel [18], and Australia [23]. Leading causes of death identified in this study were similar to those described in previous studies [24]–[25]. Likewise, our observation that males are at greater risk of death has also already been well-documented in the literature [26]–[27]. However, the main finding of our study was that clients who receive methadone treatment over extended periods and HIV-positive clients who receive ART both experienced improved survival in this setting.

In a 15-year follow-up study in Israel, a lower mortality rate was observed among DUs who stayed in MMT at least one year, compared with those who quit within one year [25]. In another study, no deaths were observed within 4 years of follow-up among subjects were enrolled in MMT. However, 17 subjects who discontinued MMT during the four year study died, representing an untreated mortality rate of 20 per 1,000 PY [23]. These are just two of the many studies that have documented improved outcomes, including survival, among DUs around the world who are retained for long periods in MMT programs. Our results support these findings and show a dramatic effect of longer-term MMT participation on mortality rates, 8.2 per 1,000 PY for four or more years in treatment and 21.7 per 1,000 PY for two to 3 years in treatment, compared to 81.1 per 1,000 PY for one year of treatment or less.

While China's MMT program has scaled-up quickly and coverage has significantly improved in recent years, client retention remains a problem. Drop-out rates of greater than 50% program-wide were reported in 2007 [9], and again in 2010 [10]. Reasons for low retention have been speculated to be overall low methadone doses, clinic accessibility, treatment interruption caused by incarceration, lack of psychosocial support within MMT clinics, concurrent drug use, and misconceptions of clients and providers about methadone treatment goals [28]–[29]. One recent study documents nearly half of MMT clients actively engaging in concurrent opiate use, the risk of which increased as treatment duration increased [12]. Additionally, in two related studies in Guangzhou, drop-out rates were again reported to be greater than 50% in 2012, and misconceptions about methadone treatment were found to predict both drop-out and poor adherence. In these studies, the majority of clients believed that MMT was a detoxification program that could be completed in two to three months and any longer-term methadone use would be harmful [28]–[29].

Some studies have found low methadone doses to be associated with higher mortality of MMT clients [19]. In this study, we identify a statistically significant association between higher methadone dose (>60 mg/day) and mortality. A majority of deaths observed in this study were among patients receiving average doses of 60 mg/day or less (129 of 154 deaths). Low methadone dose has been associated with high drop-out rates in other countries and has been speculated to be the root cause of low retention in China as well [30]. Thus, it could be that low methadone dose affects MMT retention in our sample, perhaps thereby indirectly affecting mortality. However, further studies on this issue are needed as methadone dosing practices are a very complex issue in China with provider and patient-level barriers to increased doses as well as long-term treatment [30].

We found not only that HIV-positive subjects had a higher risk of death than HIV-negative subjects in our cohort, but also that HIV-positive subjects not receiving ART had an even higher mortality rate. Although there is evidence that MMT program prevents new HIV infections and reduces HIV/AIDS-related deaths worldwide [31]–[34], and although China's National Free ART Program offers ART services free of charge to all HIV-positive citizens [35], China's HIV-positive DUs are known to have the lowest the treatment coverage and poorest outcomes, compared to all other high-risk groups [36]. Barriers to treatment at the patient level include perceptions of intolerable side effects or inability to adhere to prescribed ART regimens, comorbid psychiatric illness, addiction-related instability, and lack of health insurance. Among providers, lack of skills and experience treating co-existing drug addiction and HIV-infection is common and stigma associated with drug users and HIV-positive individuals is still very prominent [37].

Because HIV-positive MMT clients are regularly in contact with the health system for daily administration of methadone, MMT clinics offer a unique opportunity to improve ART coverage and adherence and thereby reduce mortality rates among this high-risk population. Therefore, we propose development of a “one-stop shop” model for delivery of integrated MMT and ART services for HIV-positive drug users in China. An emerging body of research in other countries has demonstrated that MMT clinic-based ART service is an effective strategy to promote treatment initiation and adherence, and at the same time enhance clinical outcome for HIV-positive DUs [38]–[43]. In the US, findings from a 12-month follow-up study indicate that HIV viral loads of <400 copies/mL were achieved by 56% of patients receiving integrated MMT and ART services [44]. While, this integrated model must be tested via controlled clinical trials, and improving outcomes for HIV-positive DUs will also require ongoing counseling on high-risk behaviors and the importance of therapy adherence, it appears that moving in the direction of this integrated model has great potential for saving lives and improving quality of life.

This study has several limitations. Most importantly, because this is an observational study, no conclusions about causality can be made. In addition, although our multivariate analyses adjusted for the key behavioral predictors of mortality, there may have been other unmeasured confounders that were not controlled. Much of the data on socio-demographics and drug use history are self-reported by drug users upon entry into the MMT program. Therefore, exposures may be under- or overestimated due to social desirability or recall bias. Similarly, missing data resulting from refusal of clients to answer more sensitive questions (*i.e.*, whether they have committed a crime in the past month) may also contribute to under- or overestimation of exposures. Finally, there were 17.3% (261) clients who lost to follow-up during the study period. It's not able for us to identify deaths among individuals lost to follow-up, which could lead to the mortality rates reported either underestimated or overestimated. The lost to follow-up would bias the study result to the opposite direction if individuals were lost because they were likely to be employed, HIV negative, and less frequent drug use per day. In addition, this study is based on the national MMT program for which it is very difficult to separate the study from the program. One issue of ethics concerns of subjects who have little or no other opportunity to access methadone but for participation in research studies like this one. At the design of pilot national MMT program in China, it is considered that timely evaluation of MMT program would provide critical information for Chinese government to consider scale-up MMT program and the data collected in the initial pilot project phase did served the purpose. Since then, clients apply for MMT program voluntarily give their consents that after removing their personal identifications their data can be used for collectively data analysis for study purposes. This does not create any harm to any individual who participate in MMT program but gives them opportunity for making contribution to sciences as well as to society.

In summary, specific interventions aimed at decreasing mortality rates among China's MMT clients are needed. The results of our study support development and implementation of interventions that will increase retention of DUs in MMT and expand coverage of ART for HIV-positive DUs. Evidence from this and other studies call for integration of MMT and ART services into a “one-stop-shop” model, so that DUs can access and engage in the care they need earlier and more consistently, thereby extending and improving the quality of their lives.
